# The other immuno-PET: Metabolic tracers in evaluation of immune responses to immune checkpoint inhibitor therapy for solid tumors

**DOI:** 10.3389/fimmu.2022.1113924

**Published:** 2023-01-09

**Authors:** Jelena Levi, Hong Song

**Affiliations:** ^1^ CellSight Technologies Incorporated, San Francisco, CA, United States; ^2^ Department of Radiology, Stanford University, Palo Alto, CA, United States

**Keywords:** Metabolism, T cells, Immunotherapy, Treatment response, PET

## Abstract

Unique patterns of response to immune checkpoint inhibitor therapy, discernable in the earliest clinical trials, demanded a reconsideration of the standard methods of radiological treatment assessment. Immunomonitoring, that characterizes immune responses, offers several significant advantages over the tumor-centric approach currently used in the clinical practice: 1) better understanding of the drugs’ mechanism of action and treatment resistance, 2) earlier assessment of response to therapy, 3) patient/therapy selection, 4) evaluation of toxicity and 5) more accurate end-point in clinical trials. PET imaging in combination with the right agent offers non-invasive tracking of immune processes on a whole-body level and thus represents a method uniquely well-suited for immunomonitoring. Small molecule metabolic tracers, largely neglected in the immuno-PET discourse, offer a way to monitor immune responses by assessing cellular metabolism known to be intricately linked with immune cell function. In this review, we highlight the use of small molecule metabolic tracers in imaging immune responses, provide a view of their value in the clinic and discuss the importance of image analysis in the context of tracking a moving target.

## Introduction

Not a new concept in cancer treatment (1), immunotherapy has gained the well-deserved attention because of the remarkable clinical outcomes achieved in a subset of patients with advanced solid tumors who previously had very limited treatment options (2). Immunotherapy that encompasses various treatment strategies - cancer vaccines, oncolytic viruses, cytokine and cell therapy, immune checkpoint inhibitors (ICI) - aims to augment natural immune responses to eliminate malignancy. Immune checkpoint inhibitors (ICI), monoclonal antibodies targeting immune checkpoints, such as programmed death-1 (PD-1), its ligand (PD-L1) (3), or cytotoxic T lymphocyte antigen-4 (CTLA-4), disrupt immune tolerance by blocking regulators of T cell activation and allowing effective anti-tumor response (4). The demonstration of the efficacy of ipilimumab (targeting CTLA-4) in metastatic melanoma (5) and its subsequent regulatory approval in 2011, led to a rapid expansion in the use of ICIs in immunooncology in a number of tumor indications and unprecedented tissue/site-agnostic authorization. The number of T cell-targeted immunomodulating therapies under development is steadily increasing as is the number of clinical trials evaluating them, reaching over 4000 investigations in 2020 (6).

The remarkable clinical successes achieved with ICIs have been met with several challenges stemming in large part from a mechanism of action that is different from conventional cytotoxic drugs and lack of predictive biomarkers of response (7). Unique patterns of response to ICIs, such as pseudoprogression and hyperprogression, represent a difficult conundrum that confounds treatment decision process for clinicians (8). Pseudoprogression, observed since early clinical trials, is a transient increase in tumor size followed by tumor regression. In pseudoprogression, the initial increase in tumor size, caused by intratumoral immune cell infiltration and inflammation, signifies a good response to therapy that would require treatment continuation past the apparent progression phase. On the other hand, a more recently described phenomenon of rapid tumor growth, hyperprogression, would necessitate immediate cessation of ICI treatment and alternative therapies. Updated radiological response criteria, such as iRECIST, were developed to address the response patterns unique to immunotherapeutics, but only assess changes in the tumor burden (9). Although tumor rejection is the ultimate goal of immmunotherapies, tumor-centric approach to response assessment neglects effects of immunotherapy on its intended targets - immune cells. By being target-focused, immunomonitoring methods have the potential to provide: 1) better understanding of the drugs’ mechanism of action and treatment resistance, 2) earlier assessment of response to therapy, 3) patient/therapy selection, 4) evaluation of toxicity and 5) more accurate end-point in clinical trials. Multiple sophisticated technologies, both tissue- and blood-based, have been developed that aid in immune contexture phenotyping (10). In this review we focus on the Positron Emission Tomography (PET) imaging biomarkers and the role they play in assessing immune response to ICI. PET imaging agents that target key players of the immune response could offer a powerful noninvasive tool for a holistic, whole-body evaluation of complex immunologic processes and simultaneous assessment of both on- and off-target effects. Although Immuno-PET traditionally refers to the use of radiolabeled monoclonal antibodies and their fragments, here, we highlight small molecule metabolic tracers and their application in imaging immune responses. We also provide a view of the value of these imaging agents in the clinic and discuss the challenges of image analysis associated with tracking a moving target.

## Imaging targets

Compelling evidence supports the significance of immune contexture, both before and during therapy, in the clinical outcome of cancer patients (11). Of all the immune cells frequently found in the tumor microenvironment, the presence of CD8+ cytotoxic T cells has been found to have the most significant positive predictive value on the patient survival. The positive predictive value of CD8+ cytotoxic T cells was found across 17 solid cancer types, including colorectal, breast, melanoma, lung, head and neck and others (12). Because of the vital role that the CD8+ T cells play in therapy response and clinical outcome, imaging agents that are being developed for non-invasive immunomonitoring aim to characterize different aspects of the CD8+ subset – their abundance (13, 14), activation (15, 16) or effector function (17). The degree of specificity for CD8+ T cells among the metabolic tracers that are the focus of this mini review, varies depending on what metabolic pathway they target (18). As the contributions of other immune subsets, in particular CD4+ T cells, on antitumor immune response is increasingly being recognized (19), imaging agents that, in addition to CD8+, assess other immune subtypes as well may be of great value for assessment of immune response to immunotherapies.

## Metabolism and T cell function

Cellular metabolism is intricately linked to T cell function, dynamically adapting to support all aspects of T cell response: activation, proliferation, survival and effector function (20). As they exit quiescence spurred by immunologic and microenvironmental cues, T cells undergo metabolic reprogramming that involves diverse changes and upregulation of glycolysis, glutaminolysis, amino acid metabolism, fatty acid oxidation and synthesis, and mitochondrial metabolism and biogenesis. This metabolic rewiring supports not only the increased demands for bioenergy, but also for biomass and production of effector molecules.

**Figure 1:**
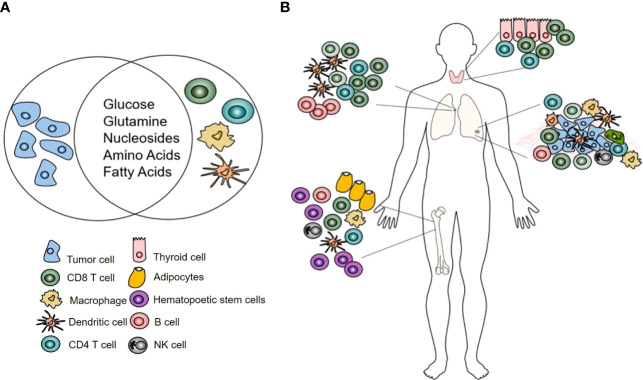
**(A)** Highly proliferating tumor and immune cells share metabolic needs. **(B)** The effects of immunotherapy on immune cells using metabolic tracers can be assessed in the tumor microenvironment but also at sites where no metabolic competition with tumor cells exists: the bone marrow, lymph node, thyroid.

The transformation of normal cells into highly proliferating, dysregulated cancer cells is also supported by alterations in metabolism. The similarities in metabolic needs and pathways employed by both activated immune cells and cancer cells results in a metabolic competition within the tumor microenvironment (21). The metabolically hostile tumor microenvironment is now recognized as one of the key mechanisms of impaired antitumor immunity and is being targeted in novel immunotherapeutic approaches (22). For metabolic tracers, the overlap between cancer cells and activated immune cells represents a significant challenge as it affects the specificity of the agents (**Figure 1**). However, despite this confounding factor, metabolic tracers have shown utility and great potential in evaluating and predicting response to ICI.

## Upregulated glycolysis

Increased uptake of glucose and accompanying lactate production were one of the earliest recognized hallmarks of cancer metabolism. A radiolabeled analog of glucose, ^18^FDG, that reports on utilization of glucose in tumors is routinely used in diagnosis, staging and restaging of oncologic patients. Because of its widespread use in oncology, the utility of FDG in assessment of response to ICI has been the most studied of all metabolic tracers. The majority of the studies, focusing primarily on melanoma and non-small lung cancer, aimed to evaluate therapy induced changes in the tumor metabolism. These investigations resulted in an evolving set of PET specific criteria that have been developed to address response patterns observed with immunotherapy (23).

As shared avidity for glucose between immune and tumor cells impedes the analysis of FDG uptake in the immune cells within the tumor microenvironment (TME) (24), a few studies that explored the use of FDG as an agent for assessment of the immune, rather than tumor response to therapy focused on examining post-therapy signal changes in non-tumor tissue. To identify the metabolic patterns induced by a successful antitumor immune response, Schwenck et al. retrospectively analyzed changes in FDG uptake in the primary and secondary lymphoid organs, specifically bone marrow and spleen, in metastatic melanoma patients treated with ICI (25). While no differences in FDG uptake were found in the spleens, the post-therapy signal change in the bone marrow showed significant differences between responders and non-responders. Post immunotherapy, responding patients showed an increase in FDG signal in the axial skeleton, while in non-responders the signal decreased. The authors suggested that the observed increase in FDG signal in the bone marrow post-therapy might indicate an increase in hematopoiesis argued to be necessary for successful immunotherapy. In addition to the change in the FDG signal post therapy, the analysis also revealed a higher baseline signal in the bone marrow of the responders. This finding seems to be in contrast to the studies that found higher FDG activity in the bone marrow to be inversely correlated with survival (26–28). A systematic review that included studies totaling more than 2500 patients suggested a direct relationship between the tumor and bone marrow glucose metabolism and systemic inflammation. The inverse association of bone marrow metabolism with survival could indicate immunosuppression associated with host systemic inflammation (27).

**Figure 2:**
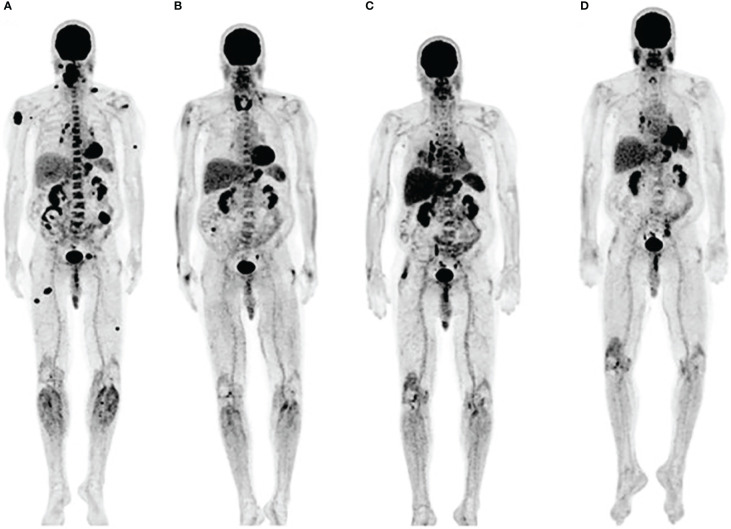
Immune related thyroiditis. **(A)** FDG scan of a metastatic melanoma patient prior to starting immunotherapy **(B)** Three months into anti-PD-1 therapy, FDG uptake in multiple metastatic lesions decreased while the signal in thyroid increased. Bilateral hilar adenopathy that developed after 6 months **(C)**, disappered at 9 months **(D)**. Adapted with permission from Wolters Kluwer Health, Inc. under the terms of CC-BY-NC-ND license: Nobashi T, Baratto L, Reddy SA, et al. Predicting Response to Immunotherapy by Evaluating Tumors, Lymphoid Cell-Rich Organs, and Immune-Related Adverse Events Using FDG-PET/CT. Clin Nucl Med. 2019;44:e272-e279. https://journals.lww.com/nuclearmed/pages/default.aspx.

In addition to evaluation of lymphoid tissue, another approach to assessing immune response using FDG is to analyze off-target toxicity associated with excessive immune reaction. Immune-related adverse effects (irAEs) can affect any organ but are most commonly observed in the gastrointestinal tract, endocrine glands, skin and liver (29). As some studies found irAEs to be associated with a better response to immunotherapy, presumably by indicating an immune flare necessary for an antitumor effect, Nobashi et al. investigated whether FDG-detectable irAEs could be a favorable prognostic marker (30). The retrospective analysis involving 40 patients with different types of cancer treated with check point inhibitors found that approximately 82% of patients with FDG-detectable irAEs had a complete response on the final restaging scan. Interestingly, patients who had an increase in FDG signal in the thyroid early in the therapy, had the greatest clinical benefit from the therapy (**Figure 2**). Those results suggested that thyroiditis, generally appearing within first weeks after the start of immunotherapy, has a potential to serve as an early indicator of immunotherapy efficacy. The recent investigations of autoimmune thyroid disease revealed the role of PD-1/PD-L1 mechanism in maintaining the immune tolerance in the thyroid and suggested that the disruption of the PD-1/PD-L1 pathway with ICIs may cause the loss of the tolerance and development of thyroiditis (31).

**Figure 3:**
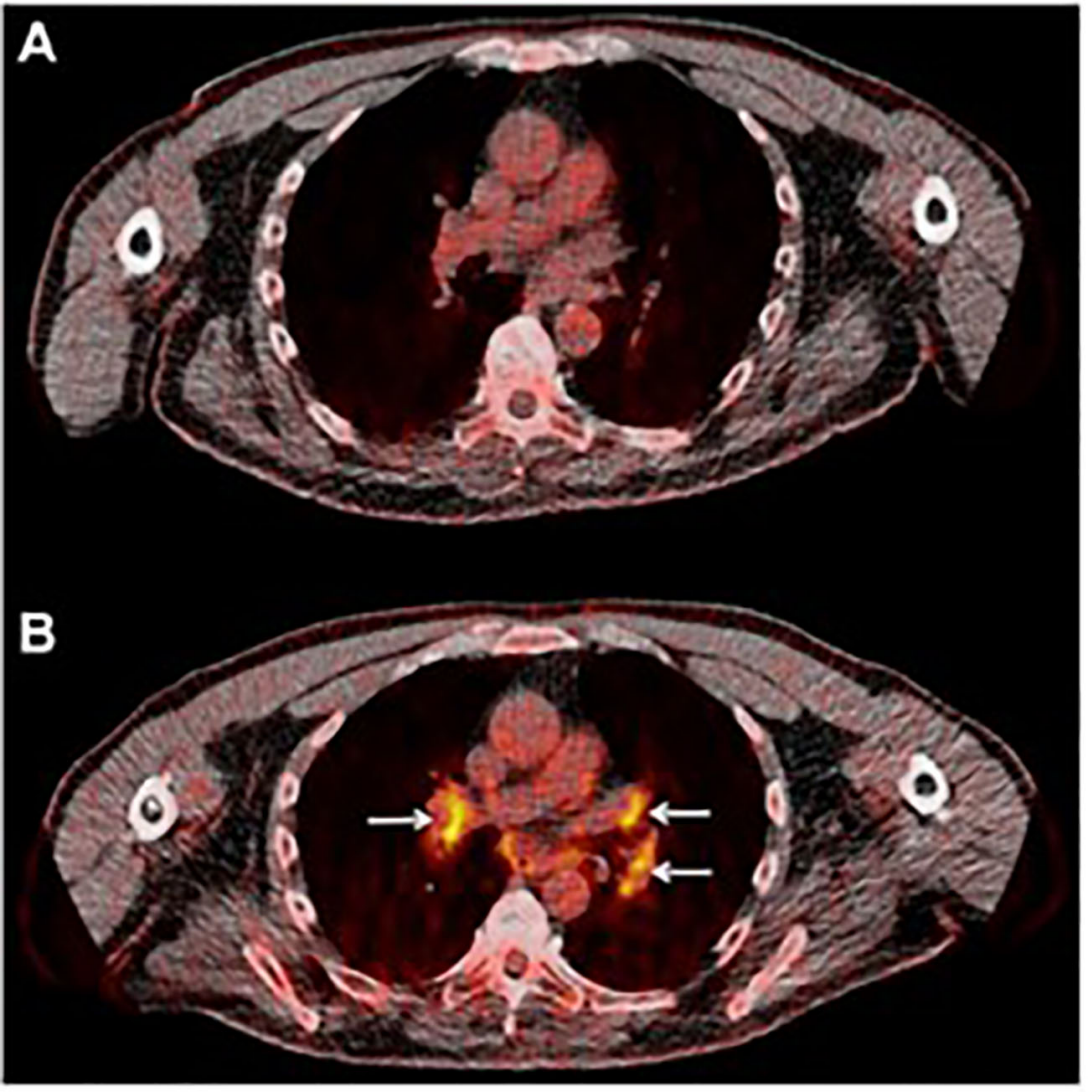
Sarcoid-like lymphadenopathy. **(A)** Transaxial FDG image of the thorax of a metastatic melanoma patient prior to starting immunotherapy **(B)** After two cycles of anti-CTLA-4 therapy FDG avidity in the mediastinal and hilar lymph nodes was evident. Adapted with permission from Springer Nature:Sachpekidis C, Larribere L, Kopp-Schneider A, Hassel JC, Dimitrakopoulou-Strauss A. Can benign lymphoid tissue changes in (18)F-FDG PET/CT predict response to immunotherapy in metastatic melanoma? Cancer Immunol Immunother. 2019;68:297-303.

Sarcoid-like lymphadenopathy, a symmetric nodal FDG uptake pattern akin to the one observed in sarcoidosis, has been noted in about 5% of patients treated with check point inhibitors (30). Also suggested to represent an immune flare response that could indicate antitumor immune activity, sarcoid-like lymphadenopathy was investigated by Sachpekidis et al. in 41 patients with unresectable melanoma treated with ipilimumab (anti-CTLA-4 antibody) (32). An increased, symmetrical FDG uptake in the mediastinal and hilar lymph nodes was observed either in the interim (after 2 cycles of ipilimumab) or at the end of the treatment (after 4 cycles of ipilimumab) in 10% of the patients (**Figure 3**). Interestingly, all patients with this radiological finding demonstrated disease control, indicating its association with response to therapy rather than disease progression. However, the lack of this characteristic nodal uptake did not imply resistance to therapy as 27 responding patients did not show a sarcoid-like reaction.

Considering the sheer volume of FDG scans, the studies that focus on the clinical value of inflammation in tissues other than tumors can bring better understanding of response to immunotherapies. Although relatively small in scope, these studies suggest the significance of off-target inflammation as an indicator of systemic immune response. Validation of these results in larger cohorts of patients is needed and prudent.

## Increased biosynthesis

Heightened proliferation, observed both in cancer and stimulated T cells, imposes needs for an increase in cellular biomass and genome replication. The biosynthetic requirements for proteins, lipids and nucleic acids in proliferating cells consequently result in amplified demand for their building blocks, amino and fatty acids, and nucleotides. Imaging agents based on these building blocks can thus assess the changes in biogenesis that occur in cancer and immune cells post therapy.

## Nucleic acid synthesis

Nucleotide pools, critically important for cells genomic stability, growth, proliferation and survival, are intricately controlled by the *de novo* and salvage nucleotide networks (33). Salvage nucleotide pathway, occurring both in the cytosol and mitochondria, recycles pre-formed nucleic bases, nucleosides and nucleotides and is more energy-efficient than the *de novo* pathway. In proliferating tissues, lymphoid organs and the brain, the salvage pathway is the critical source of nucleotides (34, 35). This reliance on salvage pathway in lymphoid tissues motivated the development of radiolabeled nucleosides for imaging immune activation.

**Figure 4:**
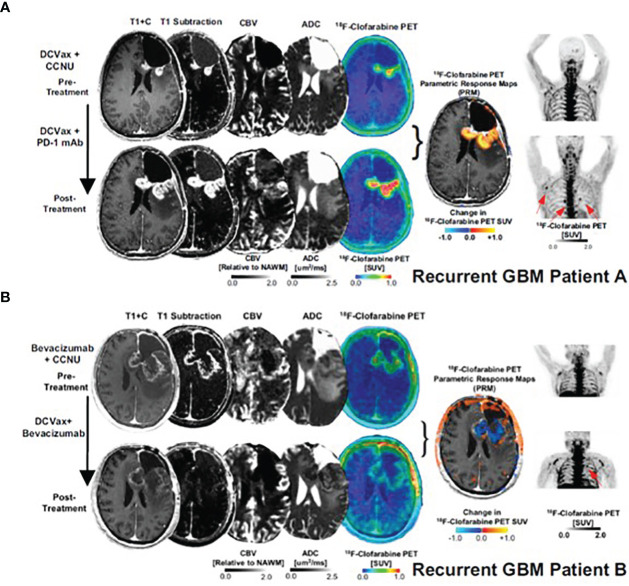
[18F]CFA PET and advanced MRI fusion images of two GBM patients **(A, B)** treated with dendritic cell vaccination and anti-PD-1 therapy.

The first agent designed to image immune cell activation, [^18^F]FAC (1-(2’-deoxy-2’[18F] fluoroarabinofuranosyl) cytosine), is a substrate for deoxycytidine kinase (dCK) in the salvage pathway and was reported by Radu et al. in 2008 (36). In preclinical models, [^18^F]FAC showed better selectivity for lymphoid organs than FDG and allowed visualization of antitumor immune response in the spleen and lymph nodes. However, [^18^F]FAC’s clinical use was precluded by its rapid deamination *in vivo*, particularly in humans. [^18^F]CFA (2-chloro-2′-deoxy-2′-[18F]fluoro-9-β-D-arabinofuranosyl-adenine), a next-generation, metabolically stable, dCK agent, showed accumulation in the lymphoid organs, bone marrow, spleen and the axillary lymph nodes, in healthy volunteers (37). Its utility in detecting immune response was assessed in recurring glioblastoma (GBM) patients after dendritic cell (DC) vaccination with and without anti-PD-1 treatment (38). Functional PET imaging with [^18^F]CFA was used in combination with advanced magnetic resonance imaging (MRI) to differentiate between tumor progression and pseudo-progression, a clinical challenge that complicates GBM patient management. An increase in posttreatment [^18^F]CFA signal was noted in the peripheral lymph nodes in two patients, with different intratumoral tracer uptake (**Figure 4**). As the study included only two patients, the clinical significance of the observed changes could not be established.

The selectivity of [^18^F]FAC for immune cells motivated the development of [^18^F]F-AraG as an agent for imaging activated T cells (39). [^18^F]F-AraG is a ^18^F-labeled analog of 9-β-D-Arabinofuranosylguanine (40), a compound that has shown selective accumulation in T cells (41, 42) and whose prodrug, nelarabine, is FDA-approved for treatment of patients with T cell acute lymphoblastic leukemia and T cell lymphoblastic lymphoma (43). A substrate for deoxyguanosine kinase (44), a kinase in the salvage pathway present solely in mitochondria and critical in supplying nucleotides for mitochondrial DNA (mtDNA) synthesis (37, 45, 46), [^18^F]F-AraG’s ability to visualize activated T cells lies in its association with mitochondrial biogenesis (47). In response to activation, T cells undergo metabolic reprogramming and dramatically increase both mitochondrial mass and mtDNA (48, 49) resulting in an increased demand for nucleotides. [^18^F]F-AraG’s specificity for T cells over tumor cells (15, 47, 50), a rather rare characteristic in metabolic tracers, comes from the interplay of enzymes in the salvage pathway, most notably dGK and sterile alpha motif and HD-domain containing protein 1 (SAMHD1), a key regulator of nucleotide pools (47, 51).

**Figure 5:**
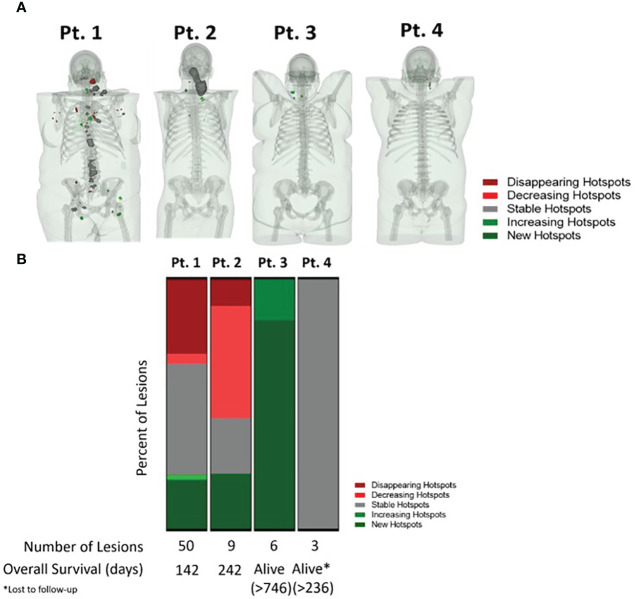
AI-assisted whole body evaluation of the change in [18F]F-AraG signal in head and neck squamous cell carcinoma patients. **(A)** The change in SUV mean in patients pre and post anti-PD-1 therapy. Different colors represent positive (>30% increase in signal, green), negative ( >30% decrease in signal, red) and no change in signal post therapy (within ± 30% signal change range, grey). **(B)** The [18F]F-AraG signal change post anti-PD-1 therapy trended with patients’ outcome. The patients with areas where the signal disappeared or decreased post therapy, indicative of the lack of T cell activation, had shorter overall survival than the patients with areas of stable and increasing signal.

Preclinically, [^18^F]F-AraG has been used to evaluate T cell involvement in graft versus host disease (GVHD) (50), rheumatoid arthritis (52), and multiple sclerosis (53). In immunoncology, preclinical studies confirmed [^18^F]F-AraG’s specificity for activated T cells and utility in predicting response to immunotherapy (15), as well as in patient/therapy selection and assessment of immune priming therapies (54). The ability of [^18^F]F-AraG to image T cells activation and thus provide early indication of adaptive response to immunotherapies in cancer patients is currently being investigated in multiple Phase II trials. An AI-assisted whole body evaluation of the change in [^18^F]F-AraG signal in four head and neck squamous cell carcinoma (HNSCC) patients after a single dose of anti-PD-1 antibody, revealed both inter and intra-patient heterogeneity in immune response to therapy (55). Importantly, the change in [^18^F]F-AraG signal trended with the clinical outcome. The patients with areas of stable or increasing signal post therapy had longer survival than patients with disappearing or decreasing hotspots (**Figure 5**). As different metastatic sites within a patient have distinct immune contextures that affect their growth and response to immunotherapy (56), quantification of a system-wide immune response treatment response could offer a more comprehensive assessment of response and provide an opportunity for therapy modification to avoid resistance and optimize patient outcome.

As a cell proliferation tracer, a deoxy thymidine analog, [^18^F]FLT, has shown promise in assessing tumor response to various standard therapies, such as chemo- and radiotherapy in different types of cancer (57). Because [^18^F]FLT accumulates both in proliferating tumor and immune cells (58), the assessments of the immune responses to immunotherapy using [^18^F]FLT were focused not on the tracer accumulation in the tumor but in the lymphoid tissues, namely in the proximal lymph nodes (59) and in the spleen (60). Intranodal vaccination of melanoma patients with antigen loaded DCs led to a markedly increased [^18^F]FLT signal in the treated LNs, but not in control LNs (59). Interestingly, the signal in the treated LNs correlated with the level of antigen-specific antibodies and proliferation of T cells in peripheral blood. Treatment of metastatic melanoma patients with anti CTLA-4 antibody, resulted in significant increase in [^18^F]FLT signal in the spleen of some patients, at a median of two months after the start of therapy (60). Unlike with [^18^F]FLT, immune reactivity could not be followed with FDG after either the DC vaccination or anti-CTLA-4 treatment.

## Upregulated lipogenesis

Alterations in lipid metabolism in proliferating cells supports their increased need for energy and cell membrane synthesis. Imaging agents that evaluate lipid metabolism alterations have largely been developed with the purpose of diagnosis of those cancers in which FDG shows limited value, such as in prostate or brain cancer. Although some of these agents accumulate in inflammation as well, their use in assessing immune responses have not been investigated (61). An increased accumulation of [^18^F]-Fluorocholine, a promising agent that reflects upregulation of cell membrane synthesis, has been observed in the non-metastatic mediastinal, axillary, inguinal and cervical lymph nodes in about 6.3% of prostate cancer patients (62, 63). Considering the uptake in other inflammatory processes involving T lymphocytes and activated macrophages (64), as well as the recent report that suggests a close relationship between PD-L1 immune checkpoint, choline metabolism and inflammation (65), it would be interesting to study whether the lymphoid tissue uptake of [^18^F]-Fluorocholine or other imaging agent that evaluates lipid metabolism might provide useful information on immune response to ICI therapy.

## Amino acid metabolism and peptide synthesis

Amino acid-based PET tracers can measure increase in protein synthesis and amino acid transport in highly proliferative cells (66). To date, primary focus of investigations involving radiolabeled amino acids has been their utility in brain tumor imaging (67). Despite high demand for amino acids in effector T cells and upregulation of amino acid transporters during T cell activation (68), no studies as yet assessed the value of amino acid PET agents in evaluating immune response to immunotherapy.

## Imaging-based treatment response in the clinic

It is estimated that about 40% of cancer patients in the United States are eligible for ICIs (69). Other novel immunotherapies such as CAR-T cell therapy have also been increasingly adopted clinically. As a result, imaging-based evaluation of treatment response has become more and more important in clinical practice. The main goal of current imaging-based treatment response evaluation is identification of patients who do not respond to the immunotherapy regimen and may benefit from change of treatments as early as possible. Traditionally, lesion size-based response criteria, RECIST 1.1, has been the most widely used for evaluating treatment response. However, the change in anatomic size typically occurs relatively late after treatment and RECIST 1.1 does not consider the metabolic changes after treatment, an early tumor response biomarker. FDG PET based PERCIST (70) and EORTC (71) criteria were thus proposed to incorporate metabolic changes to assess treatment response. Other widely adopted metabolism-based treatment criteria include Lugano Classification for lymphoma treatment response. However, the observed atypical patterns of tumor response after ICIs, such as pseudoprogression and hyperprogression, pose challenges even to these, FDG metabolism-based criteria of treatment response. Several new criteria have therefore been proposed to evaluate treatment response specifically for immunotherapy. irRECIST (72) and iRECIST (9) are lesion size based and modified from RECIST 1.1. PECRIT (73) and PERCIMT (74) are based on both size and metabolic changes and modified from RECIST, PERCIST and EORTC. The main changes in these new criteria include a confirmation scan in 4-12 weeks for evaluating pseudoprogression and different approaches to incorporate new lesion measurements.

In addition to the interim and end-of-therapy imaging-based treatment assessments, pretherapy imaging that can predict treatment response and toxicities would be invaluable for immunotherapies. For many cancers treated with immunotherapy, durable responses are observed only in about 20% patients (75) while severe immune-related adverse effects (irAEs) could be seen in up to 10-15% patients with mortality ranging from about 0.4% in patients treated with single agent to 1.2% in combined therapy (76). Another undesirable outcome associated with immunotherapy is hyperprogression, the incidence of which varied from 5.9% to 43.1% based on a meta-analysis of 3109 patients (77). The mechanism of hyperprogression is poorly understood and difficult to predict. Pretherapy scans with predictive value could help improve response rates and avoid severe immunotherapy induced side effects and poor outcome due to hyperprogression. PD-L1 immunohistochemistry assays have been used clinically as a predictive tumor biomarker to determine whether patients will benefit from ICIs. However, the level of expression can vary between different cancer types, specific assay types and more importantly, PD-L1 expression can be heterogeneous at different locations. While it is unrealistic to biopsy many lesions at multiple time points, non-invasive PET imaging can offer a whole-body assessment of PD-L1 expression levels. Indeed, ^89^Zr labeled anti-PD-1 PET radiotracer ^89^Zr-pembrolizumab (78), ^89^Zr-nivolumab (3) and anti-PD-L1 radiotracer ^89^Zr-atezolizumab (79) have shown correlation between uptake and treatment response. Interestingly, ^89^Zr-pembrolizumab tumor uptake did not correlate with PD-L1 or PD-1 immunohistochemistry (3).

The majority of patients treated with ICIs have mild irAEs and can continue treatment under monitoring while patients with moderate to severe irAEs (Grade 2 and higher) may require suspension of treatment and possible corticosteroid treatment (80). Incidence, involved organs, severity and onset depend on cancer types and ICIs received. The onset of irAEs varies significantly with a median onset of approximately 40 days (81). The main goal of imaging is to aid early identification of irAEs and treatment. While most irAEs are monitored clinically, imaging can be helpful in the workup, which include CT chest for pneumonitis, CT abdomen and pelvis for colitis and its complications, adrenal CT for metastases or hemorrhage, CTPA or VQ scan for venous thromboembolism, MR brain for hypophysitis, aseptic meningitis, encephalitis or demyelinating disease, MR spine for Guillain-Barré or peripheral neuropathy, cardiac MR for myocarditis, MR for myositis and MR of affected joints to differentiate arthritis, metastasis or septic arthritis (80). While many of the inflammatory findings related to irAEs can be visualized on FDG PET/CT, whether metabolic changes correlate with clinical symptoms or severity of irAEs remains to be verified (82). FDG PET/CT has not been incorporated in routine clinical practice for irAEs workup.

PET imaging tracers that target components of the immune system itself in addition to tumor response could increase specificity of imaging findings after immunomodulating therapy, especially when these imaging signals can be quantified. Compared to antibody and fragment-based agents, the small molecule radiotracers targeting altered T cell metabolism typically have better tumor penetration and fast plasma clearance and thus allow patient-convenient same day imaging. In addition, these small molecule radiotracers make it possible to better evaluate systemic immune activation in the setting of ICI therapy and therefore potentially increase accuracy of predicting response and aid in the early detection of severe irAEs.

## Image analysis considerations

Taking into consideration association between immune activation, irAEs and tumor response (83), several non-traditional image analysis approaches were investigated. Wong et al. found the increased spleen to liver ratio (SLR) on pre-treatment FDG PET is associated with poor overall survival (84). The authors postulated that failure to control tumor despite activated immune system on pre-treatment scan could attribute to the lack of efficacy after further immune stimulation by ICIs. In addition, Prigent et al. reported that a 25% increase in SLR_mean_ from baseline is correlated with poor outcome in melanoma patients treated with immunotherapy (85). It was suggested that splenic uptake is more indicative of innate immune activation that could promote tumor progression rather than T cell activation. In addition to SLR, bone marrow to liver ratio (BLR) has also been correlated with poor survival in melanoma patients treated with ICIs (28). More studies are needed to elucidate these findings for better understanding of the systemic responses needed for proper tumor control.

Abundance of tumor infiltrative T cells are associated with better tumor response (86), however, imaging these T cells with FDG PET is difficult due to increased FDG uptake in both tumors and activated immune cells in the tumor microenvironment. Several novel artificial intelligence (AI) AI based imaging analysis tools have been developed and show improved prediction of immunotherapy response based on radiomic signatures (87, 88). In addition, Tunali et al. have shown that radiomic features can predict hyperprogression in non-small cell lung cancer patients after immunotherapy (89). These radiomics-based analyses still need to be validated in larger clinical studies before they can be implemented in the clinical practice. Compared to conventional radiology and FDG PET, radiotracers targeting immune cells pose additional challenge for image analysis. Infiltrative CD8+ cytotoxic T cells are heterogeneously distributed, with large variations within the same tumor lesion and between different tumor sites (90). In addition, the dynamic nature of T cell recruitment and activation makes it important to optimize time of imaging after the start of therapy. Given these characteristics of T cells, signal quantification metrics other than the most commonly used, SUV_max_ need to be investigated (55). A closer look into the SUV_peak_, SUV_mean_, SUV based heterogeneity index, metabolic volume of the radiotracer, total lesion metabolic uptake, SLR or BLR, may be necessary to further understand the utility of metabolic radiotracers in immunotherapy.

## Conclusion and future direction

Given the link between cellular metabolism and signaling pathways, cell function and fate, tracers that assess immunometabolism may find utility in several aspects of immunooncology, from drug discovery to patient selection and assessment of immunotherapy response. Although for some tracers, the similarities in metabolism between activated immune cells and cancer cells may confound assessment of immune responses within the TME, evaluation of immune activity in non-tumor tissue provides clues on the systemic immune processes. This is of high significance as recent findings, such as the effect of microbiome on the immunotherapy response, denote the critical significance of processes outside of tumors and the need for a better understanding of the systemic immunity in cancer (91).

The key to fully capturing the ability of these agents to reveal complex immunological networks lies in the development of new, whole-body image analysis approaches. Patient-level quantitative analysis of the response is perhaps the most pressing in metastatic patients as different metastatic sites have distinct immune contextures that affect their growth and response to immunotherapy (90). Automated quantitative image analysis overcomes the impracticality of manual measurement of all sites of interest and may allow extraction of predictive information relating to systemic immune responses. The advantages and promising clinical performance of the automated platforms developed for specific tracers (92, 93) may inspire investigations of such analysis approaches for immune-metabolic tracers.

## Author contributions

JL and HS wrote the manuscript. All authors contributed to the article and approved the submitted version.
